# Causal associations between mobile phone usage and glaucoma risk: A Mendelian randomization study

**DOI:** 10.1097/MD.0000000000040666

**Published:** 2024-11-29

**Authors:** Rui Song, Yinnuo Wang, Yanbo Kong, Xinyu Fan, Chuang Yuan, Xu Zha

**Affiliations:** a Department of Ophthalmology Department, The Second Affiliated Hospital of Kunming Medical University, Kunming, China; b Department of Gastrointestinal Surgery, The Second Affiliated Hospital of Kunming Medical University, Kunming, China.

**Keywords:** glaucoma, Mendelian randomization analysis, mobile phone

## Abstract

Previous research has indicated a possible link between mobile phone usage and the incidence of glaucoma. This study employs a 2-sample Mendelian randomization (MR) analysis to examine the causal relationship between mobile phone use and glaucoma risk. We used single nucleotide polymorphisms (SNPs) from publicly accessible genome-wide association study (GWAS) datasets as instrumental variables (IVs). The primary analytical method was the inverse variance weighted (IVW) approach, with MR-Egger and weighted median analyses serving as complementary methods. Sensitivity was evaluated using Cochran’s Q test and MR-Egger regression. The results demonstrate a causal effect of mobile phone usage on an increased risk of glaucoma (OR_IVW_ = 1.358, 95% CI: 1.052–1.752, *P* = .019; OR_MR-Egger_ = 1.882, 95% CI: 0.53–6.682, *P* = .337; OR_Weighted median_ = 1.387, 95% CI: 1.012–1.900, *P* = .042; OR_MR-PRESSO_ = 1.358, 95% CI: 1.052–1.752, *P* = .026). Sensitivity analyses confirmed the robustness and reliability of these findings. The study identifies mobile phone usage as a potentially modifiable risk factor for glaucoma, providing new avenues for exploring the specific mechanisms underlying these ocular disorders.

## 1. Introduction

Glaucoma, the primary cause of permanent blindness globally, imposes a significant economic burden and is projected to affect over 100 million people by 2040.^[1]^ Primary glaucoma manifests through progressive retinal ganglion cell degeneration and characteristic visual field loss, primarily associated with elevated intraocular pressure (IOP).^[2,3]^ Accurate IOP monitoring is crucial for effective disease management. However, the unpredictable and insidious onset of glaucoma necessitates ongoing research into its genetic causes and risk factors.

The rapid adoption of mobile phones and digital smart devices presents a novel, modifiable factor in glaucoma management. Responsible mobile phone use could aid in the early detection and prevention of vision loss, potentially mitigating the burden of irreversible blindness caused by glaucoma.^[4]^ Reports indicate that young people and adolescents spend approximately 5 hours daily on mobile phones.^[5]^ Many scholars have found that excessive use of smartphones is associated with various eye diseases, such as cataracts, corneal edema, lacrimation, endothelial cell loss, and retinal degeneration.^[6,7]^ These conditions can exacerbate or contribute to the development of glaucoma. Moreover, mobile phone usage has been shown to increase IOP, further elevating glaucoma risk.^[8–10]^ Thus, definitive causative analysis is essential for healthcare providers to formulate preventative strategies and programs.

Previous observational studies have faced challenges due to potential confounding factors and reverse causality.^[8,11,12]^ Additionally, systematic, evidence-based randomized controlled trials (RCTs) linking mobile phone use to glaucoma are lacking, primarily due to the high costs and time requirements of RCTs. Mendelian randomization (MR) offers an alternative approach, leveraging genetic variations as IVs to infer causality while mitigating confounding biases and reverse causality inherent in traditional observational research.^[13]^ Grounded in Mendel’s second law, MR assumes alleles are randomly allocated, thus reducing biases from confounding factors.^[14]^ Grounded in Mendel’s second law, MR assumes alleles are randomly allocated, thus reducing biases from confounding factors.^[15]^ This study utilizes a 2-sample MR approach to investigate the causal relationship between mobile phone use duration and glaucoma, with significant public health and clinical implications for prevention and early detection.^[16]^

## 2. Method

### 2.1. Study design

**Figure 1.**
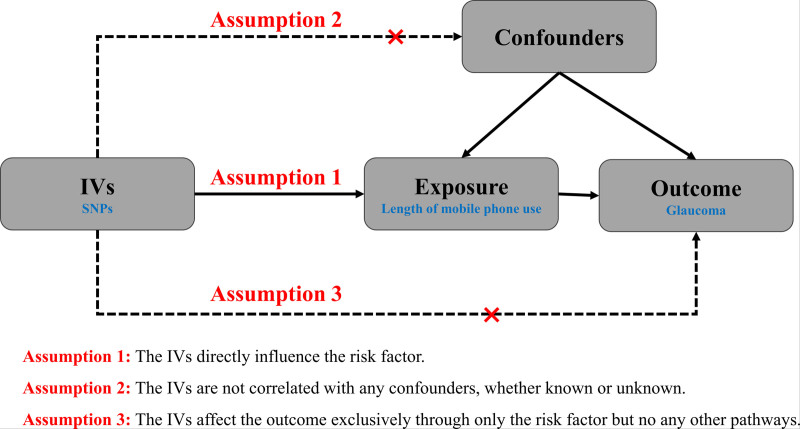
Flowchart of the study. IVs = instrumental variables, SNPs = single nucleotide polymorphisms.

This study follows a classic 2-sample MR framework, utilizing SNPs as IVs to examine the causal relationship between mobile phone usage and glaucoma risk. The exposure variable was the duration of mobile phone use, and the outcome variable was glaucoma incidence. For valid MR analysis, IVs must satisfy 3 assumptions: direct influence on the risk factor, no correlation with any confounders, and exclusive effect on the outcome through the risk factor. A flowchart illustrating this process is presented in Figure 1.

### 2.2. Data source

GWAS data on mobile phone use duration were obtained from the UK Biobank (UKB) via the Integrative Epidemiology Unit (IEU) Open GWAS Project, involving 456,972 samples and 9851,867 SNPs. Glaucoma data were sourced from the FinnGen Project (R10, December 2023), comprising 20,906 cases and 391,275 controls. These datasets, being publicly available GWAS summary statistics, did not require additional ethical approval.

### 2.3. Instrumental variable selection

A rigorous selection process ensured the robustness of IVs. Genetic variants associated with the exposure at a genome-wide significance level (*P* < 5 × 10^−8^) were identified, and SNPs correlated with the outcome were excluded. SNPs were clumped using an *r*^2^ threshold of 0.001 and a window size of 10 MB, assessed in the European 1000 Genomes reference panel. Pleiotropic SNPs were detected and excluded using the MR pleiotropy residual sum and outlier (MR-PRESSO) method. The F statistic ($F=\frac{\beta^{2}}{se^{2}}$) was calculated to ensure IV strength, with values above 10 indicating valid IVs. Ambiguous and palindromic SNPs were harmonized for accuracy.

### 2.4. Statistical analysis

A 2-sample MR approach was employed to investigate the causal relationship, with IVW analysis as the primary method. In cases of SNP heterogeneity, a random-effects IVW model was used. Complementary methods included MR-Egger and weighted median analyses. The analyses were conducted using *R* (version 4.3.1), specifically the 2-sample MR (version 0.5.7) and MR-PRESSO (version 1.0) packages.

### 2.5. Sensitivity analyses

Sensitivity analysis involved a multistep process to assess the second and third MR assumptions. Cochran’s Q test evaluated heterogeneity among IVs, while MR-Egger regression examined potential horizontal pleiotropy. Results indicated minimal heterogeneity and the absence of pleiotropy, confirmed by symmetry in the funnel plot and stability in leave-one-out sensitivity analysis.

## 3. Results

### 3.1. IV selection

A total of 29 SNPs were selected as IVs. The F-statistics for these IVs were all above 30.03 (Table 1), indicating a strong likelihood that our IV selection was not affected by weak instrument bias. This confirms that the IVs used in our MR analysis possess sufficient statistical robustness, significantly reducing the potential for bias arising from weak instruments.

**Table 1:** The SNPs selected as IVs.

SNP	Effect	Other	chr	pos	SE	pval	*F*
rs12437348	A	G	14	36,137,344	0.00314	.00080	30.03331
rs11229008	A	G	11	57,351,366	0.00595	.03357	30.22189
rs344868	T	C	2	139,338,230	0.00330	.00167	30.55295
rs10807124	A	G	6	33,436,287	0.00318	.06089	30.62280
rs7859831	T	C	9	123,443,475	0.00412	.00068	31.39267
rs11236714	T	C	11	70,578,454	0.00358	.00067	31.66064
rs853946	T	C	10	118,406,960	0.00284	.00148	31.70719
rs13266457	T	C	8	105,074,558	0.00303	.01719	31.85643
rs849527	G	A	2	205,727,801	0.00288	.02285	31.99488
rs28713780	C	T	7	3,281,783	0.00296	.00092	32.68483
rs1512142	A	G	4	47,002,288	0.00287	.00039	33.20534
rs10828247	G	A	10	21,533,927	0.00299	.00098	33.42659
rs1320650	A	T	11	115,917,589	0.00295	.00024	33.48811
rs17374152	G	A	5	93,901,242	0.00333	.00022	34.08861
rs17156711	G	A	5	104,588,998	0.00311	.00349	34.56318
rs12145998	T	C	1	205,000,291	0.00325	.00012	35.22464
rs77878475	A	T	16	17,964,691	0.00531	.00037	35.87809
rs6131703	G	A	20	15,774,039	0.00292	.00074	36.12146
rs11682846	T	C	2	156,152,191	0.00286	.00014	37.35223
rs78166132	C	T	5	161,812,980	0.00490	.00009	38.72824
rs2161220	A	G	5	80,946,085	0.00331	.00003	39.11829
rs2836920	G	T	21	39,140,992	0.00293	.00003	40.21809
rs10107145	G	A	8	10,900,703	0.00286	.00638	40.78543
rs6780051	T	G	3	56,159,122	0.00608	.00001	42.54793
rs8014346	A	G	14	46,362,799	0.00285	.00001	43.75805
rs359265	A	G	2	60,229,275	0.00292	.00001	51.71568
rs9896202	C	T	17	79,804,428	0.00285	.00000	54.13390
rs1892417	C	T	1	41,314,001	0.00340	.00002	59.32792
rs6063374	G	A	20	49,216,460	0.00343	.00004	73.42642

Abbreviations: chr = the chromosome where the SNP was located, effect = the effect allele, other = other alleles, pos = The position where the SNP was located, pval = *P* value, se = standard error.

### 3.2. MR estimate

**Figure 2.**
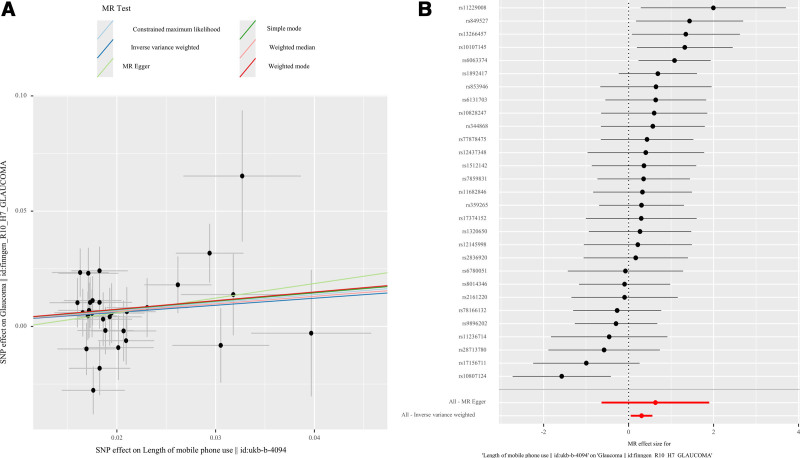
(A) Scatter plot of SNP effects. (B) Forest plot of MR effect size. MR = Mendelian randomization, SNP = single nucleotide polymorphism.

The MR analysis results indicated that the length of mobile phone use was associated with an increased risk of glaucoma. The specific values were OR_IVW_ = 1.358 (95% CI: 1.052–1.752, *P* = .019), OR_MR-Egger_ = 1.882 (95% CI: 0.53–6.682, *P* = .337), OR_Weighted median_ = 1.387 (95% CI: 1.012–1.9, *P* = .042), and OR_MR-PRESSO_ = 1.358 (95% CI: 1.052–1.752, *P* = .026) (Table 2). The MR estimates of the SNPs are illustrated in the scatter plot (Fig. 2A). Additionally, the forest plot displays each SNP’s causal impact on glaucoma (Fig. 2B).

**Table 2:** MR analysis results.

Method	β	*P* value	OR (95% CI)
IVW	0.306	.019	1.358 (1.052–1.752)
MR-Egger	0.632	.337	1.882 (0.53–6.682)
Weighted median	0.327	.042	1.387 (1.012–1.9)
MR-PRESSO	0.306	.026	1.358 (1.052–1.752)

### 3.3. Sensitivity analyses

**Figure 3.**
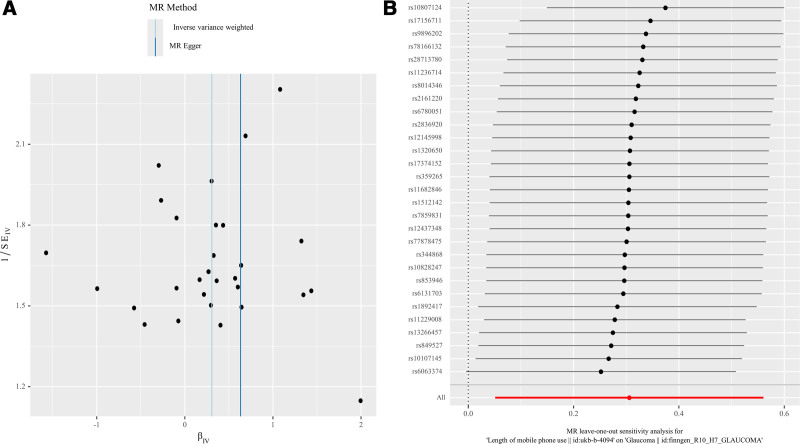
(A) Funnel plot of SNP effects. (B) Forest plot of the MR leave-one-out analysis. MR = Mendelian randomization, SNP = single nucleotide polymorphism.

Sensitivity analyses were conducted to validate the stability and reliability of our findings. The initial assessment using Cochran’s Q test revealed minimal heterogeneity among the IVs, with *P* values for the IVW and MR-Egger methods of .087 and .074, respectively (Table 3). This lack of heterogeneity was further corroborated by the symmetry observed in the associated funnel plot (Fig. 3A). Additionally, the MR-PRESSO global test and MR-Egger regression analysis yielded *P* values of .101 and .611, respectively (Table 3), indicating the absence of horizontal pleiotropy. These results suggest that the selected IVs are unlikely to affect the risk of glaucoma through mechanisms unrelated to mobile phone usage. The stability of these findings was further confirmed by a leave-one-out sensitivity analysis, where the sequential exclusion of each SNP did not significantly alter the results (Fig. 3B).

**Table 3:** Results of the sensitivity analysis.

Test	Method	Effect size	*P* value
Heterogeneity	Q_MR-Egger_	38.272	.074
	Q_IVW_	38.648	.087
Pleiotropy	Egger intercept	−0.007	.611
	Global Test	41.512	.101

## 4. Discussion

According to current understanding, Glaucoma is a group of progressive neuropathies affecting the optic nerve, resulting in the destruction of retinal ganglion cells and their axons. It is the primary cause of irreversible blindness globally. Up to 40% of patients lose their vision within the first year of diagnosis.^[17]^ Despite the severe impact, current clinical risk factors inadequately predict which patients will develop sight-threatening glaucoma.^[18]^

The advent of the information technology era has rendered mobile phones indispensable in daily life. The duration and proximity of mobile phone use during activities such as reading or calling may pose health risks. Studies have highlighted several potential adverse health effects of mobile phones, including obesity, depression, stress, and eye irritation.^[19]^ The majority of mobile phone use involves direct visual engagement, with screens directly facing the eyes, which raises concerns about excessive usage being a risk factor for various eye conditions such as digital eye strain, dry eye, myopia, and macular light damage.^[20–23]^

In this study, we further explored whether the duration of mobile phone use serves as a novel risk factor for glaucoma. Unlike prior observational studies, we employed a 2-sample MR approach, utilizing single nucleotide polymorphisms (SNPs) associated with mobile phone use duration from the UK Biobank as instrumental variables (IVs). This methodology was designed to assess the potential causal relationship between mobile phone usage and glaucoma risk. Our findings indicate a significant association between extended mobile phone use and an elevated risk of glaucoma, with consistent results across various MR sensitivity analyses.

The mechanisms by which prolonged mobile phone use may lead to glaucoma involve multiple factors. Firstly, blue light emitted from mobile phone screens is known to be harmful.^[24]^ Research has demonstrated that high-energy blue LED light from mobile phones negatively impacts mitochondrial function by reducing ATP levels and activating AIF and heme oxygenase-1, which could contribute to glaucoma development.^[25,26]^ Secondly, IOP, a major risk factor for glaucoma, has been shown to increase significantly in glaucoma patients following mobile phone use.^[8,27]^ Lee et al conducted a study involving 31 healthy participants and 127 glaucoma patients engaged in continuous fixation tasks using mobile phones, with all participants showing a significant rise in IOP.^[9]^ Another study comparing reading on mobile phones versus printed text among healthy volunteers found a greater increase in IOP with mobile phone reading.^[10]^

Additionally, myopia and dark environments may serve as intermediate links between mobile phone usage and glaucoma. Prolonged mobile phone use is associated with a higher risk of myopia.^[28]^ Numerous studies have identified both myopia and dark environments as risk factors for glaucoma, with individuals with myopia being more susceptible to glaucoma.^[29]^ Individuals suffering from myopia have a higher propensity to develop glaucoma, with high myopia exhibiting the greatest risk, followed by low and moderate myopia, which present an intermediate risk.^[30]^ Subsequent MR studies have confirmed that myopia plays a critical role in increasing glaucoma risk.^[31,32]^ Notably, the amount of time spent using mobile phones is increasing exponentially, and many people use their phones within an hour before bedtime in a dark room.^[33]^ The rising duration of mobile phone use, particularly in dark environments before bedtime, may further exacerbate this risk. Dark room provocative testing is already used to identify patients at risk for glaucoma, supporting the causal relationship between mobile phone usage and glaucoma risk through myopia and dark environments.^[34]^ These studies offer a potential rationale for the causal relationship between mobile phone usage and increased risk of glaucoma. Mobile phone usage may increase the risk of developing glaucoma by impacting myopia and the dark environment.

Our research offers several significant merits. Firstly, this study is pioneering in systematically analyzing the impact of mobile phone use duration on glaucoma incidence using MR analyses. Rigorous sensitivity analyses validated the MR assumptions, enhancing the reliability of our results. Secondly, previous observational studies faced challenges in addressing reverse causation and confounding factors such as near work, accommodative stress, dark room provocation, and high myopia.^[8,11,12]^ The MR design effectively mitigates these risks. Thirdly, the large sample size ensured sufficient statistical power and included a strict selection of IVs to meet randomization assumptions, reducing endogeneity risks and strengthening causal inference. Fourthly, to minimize population stratification bias, we utilized GWAS summary statistics from individuals of European descent.

However, our study has certain limitations. Firstly, all participants were of European descent, potentially limiting the external validity of our findings to other populations. Further investigations are required to validate our results in non-European populations. Secondly, the prevalence of different glaucoma subtypes varies by age, sex, and region. Our study did not conduct a classification analysis based on subtypes, which, while not affecting internal validity, suggests that future research should include subtype-specific MR analyses for more detailed insights.

## 5. Conclusion

In this study, we conducted a 2-sample MR analysis utilizing genetic instruments derived from extensive GWASs. The findings provide robust evidence supporting a causal relationship between prolonged mobile phone use and an increased risk of glaucoma. Understanding the specific nature of this association may pave the way for new research into the mechanisms underlying these ocular disorders, offering potential pathways for prevention and treatment strategies.

## Author contributions

**Conceptualization:** Chuang Yuan.

**Data curation:** Rui Song, Yanbo Kong.

**Funding acquisition:** Xu Zha.

**Methodology:** Rui Song, Yanbo Kong.

**Supervision:** Xu Zha.

**Writing – original draft:** Rui Song, Yinnuo Wang, Xinyu Fan, Chuang Yuan.

**Writing – review & editing:** Xu Zha, Chuang Yuan.
